# Acute Encephalopathy Workup for a Patient With a History of Lupus Cerebritis and Unremarkable Inflammatory Markers: A Case Report

**DOI:** 10.7759/cureus.51699

**Published:** 2024-01-05

**Authors:** Connor Dasbach, Doo Hee Kim, Ryan Pinti, Bibban Bant Deol

**Affiliations:** 1 Internal Medicine, Wayne State University School of Medicine, Detroit, USA

**Keywords:** lupus flare, sle and lupus encephalitis, lupus cerebritis, non inflammatory lupus, systemic lupus erythematosis

## Abstract

Lupus is a relatively rare disease; however, many of the patients diagnosed with lupus experience an acute confusional state. Despite the prevalence, lupus cerebritis remains a diagnosis of exclusion due to the number of differential diagnoses of the cause of acute confusional state and unreliable clinical markers. This case report highlights the significant duration it takes to work up a broad differential before initiating treatment for lupus. Our case involves a 65-year-old woman with a documented history of lupus, who presented with acute encephalopathy. Following the comprehensive diagnostic investigation, the patient was treated with high-dose steroids that resolved the patient’s symptoms. This report considers the option of empiric steroid treatment in the setting of acute encephalopathy in individuals with a history of lupus cerebritis with inconclusive test results.

## Introduction

An acute confusional state is common in patients with lupus, but it remains a diagnosis of exclusion due to the numerous different causes of altered mental status [1]. The pathogenesis of neurologic complications directly associated with lupus is not well understood, and inflammatory and noninflammatory mechanisms have been proposed.

The evidence for inflammatory pathogenesis is supported by the fact that there has been data showing that lupus cerebritis is associated with cerebrospinal fluid pleocytosis [2]. Many inflammatory markers have been proposed to guide clinical diagnosis, but very few have been reliable in clinical practice. The contenders based on the literature review are antiphospholipid antibodies (aPL) and anti-neuronal antibodies (anti-N) [3].

Antiphospholipid antibodies have been shown to upregulate intracellular adhesion molecule (ICAM), vascular cell adhesion molecule (VCAM), and E-selectin, leading to an increased inflammatory response and an increased risk of thromboembolic event. The thromboembolic event associated with aPL has been thoroughly studied as it relates to spontaneous abortions, and patients with lupus with positive aPL were found to have an increased risk of cerebrovascular events, seizures, headaches, and chorea [3].

A literature review by Efthimiou and Blanco showed that patients with lupus psychosis had elevated anti-neuronal antibodies. They cited studies demonstrating an eight-fold increase in immunoglobulin (Ig)G serum levels during an episode. In addition, in those patients with psychosis, there was an increased level of IgG levels compared to those not actively experiencing psychosis. The levels of IgG fell after the treatment of psychosis [3].

Other antibodies that are currently being studied are anti-ribosomal P antibodies (anti-P), anti-endothelial cell antibodies (AECA), and anti-N-methyl-D-aspartate receptor (NMDAR) antibodies, but the studies on these antibodies are conflicting and their reliability remains questionable to use in clinical practice.

Despite the inflammatory nature of lupus, the literature shows that the noninflammatory process of development for neurologic manifestations of lupus is common [4]. A study suggests that people who develop neurologic symptoms lack a healthy blood-brain barrier and are susceptible to the pathologic entry of cytokines and autoantibodies into the CNS, especially small vessels (arterioles and capillaries). Noninflammatory microangiopathy with small vessel hyalinization and associated microinfarction is a common neuropathologic finding in patients with lupus.

This case report aims to explain the complexities of lupus cerebritis by looking closely at a patient who presented to the hospital for acute encephalopathy. It focuses on the complexities of diagnosis and treatment for what appeared to be non-inflammatory lupus cerebritis and whether empiric treatment with steroids should be an option to consider in patients with inconclusive tests.

## Case presentation

The patient is a 65-year-old woman with a past medical history of lupus cerebritis, Hepatitis C, congestive heart failure, cerebral vascular event (12 years prior), seizure disorder, chronic hypertension, colon cancer, and opioid use disorder presented to the emergency department (ED) after being brought in by police after finding her confused. Her history of lupus cerebritis was noted four years prior, however, her presentation during the initial episode was not in the electronic medical record. The patient appeared well and was talkative, she was alert and oriented. However, her memory was unclear, and couldn’t formulate why she was in the ED. She had no acute concerns.

In the ED, she received a chest X-ray, EKG, and urinalysis which were unremarkable. Her initial troponin was mildly elevated, and the repeat test showed a further increase. The patient was admitted to the floor for acute encephalopathy and elevated troponins.

On admission, she had difficulty with her memory. She did not remember much of the day or how she got to the emergency room. She was able to give a general history, but she was unable to give definite details. She had chronic intermittent chest pain that she rated at 7/10, though she denied having this at the time of admission. The pain was an achy pain that did not radiate to the back. She denied any diaphoresis or shortness of breath while the chest pain occurred. She does have a history of hypertension (unmedicated last few weeks per patient), prior cerebrovascular incident, and a history of smoking 1/2 packs per day for 20 years. She also reported having headache-like pains but reported they lasted for less than a second. She endorsed chronic vision changes but nothing acute. The initial neurological exam showed she was alert and oriented to person and place, not time. Cranial nerves II-XII were intact with no focal deficits. Muscle strength, sensation, and reflexes were unremarkable. 

The patient received a complete workup for acute encephalopathy. Her B12 (438 pg/mL, normal within 180-914 pg/mL), folate (20 ng/mL, normal greater than 5.9 ng/mL), thyroid stimulating hormone (TSH) (.9 Micro IU/mL, normal within .45-5.33 Micro IU/mL), lipids (117 mg/dL, normal below 150 mg/dL), and ammonia (49 micromol/L, normal 16-53 micromol/L) were all within normal limits. Urine drug screen and syphilis screening were negative. Her medication levels were tested, and valproic acid levels came back low, indicating she may not have been taking her medications. Her parathyroid hormone (PTH) was slightly elevated, however, it only caused asymptomatic hypercalcemia (11.2 mg/dL on presentation). An EEG showed a moderate cerebral dysfunction consistent with metabolic encephalopathy or bilateral structural process, however, no active seizures. A transthoracic echocardiogram didn’t show any pericarditis, only mild mitral, aortic, and tricuspid valve regurgitation. Lumbar puncture came back with cerebrospinal fluid values that were within normal limits and polymerase chain reaction (PCR) for Herpes simplex virus (HSV) came back negative. The patient was tested for serum antiphospholipid antibodies, ribosomal P, and anti-NMDA which came back negative. The anti-nuclear antibody was minimally elevated (1:160), anti-dsDNA was negative, complement C3 was 81 mg/dL, complement C4 25 mg/dL and C-reactive protein was 5.2 mg/L which lowered active Lupus flare on the differential.

**Figure 1:**
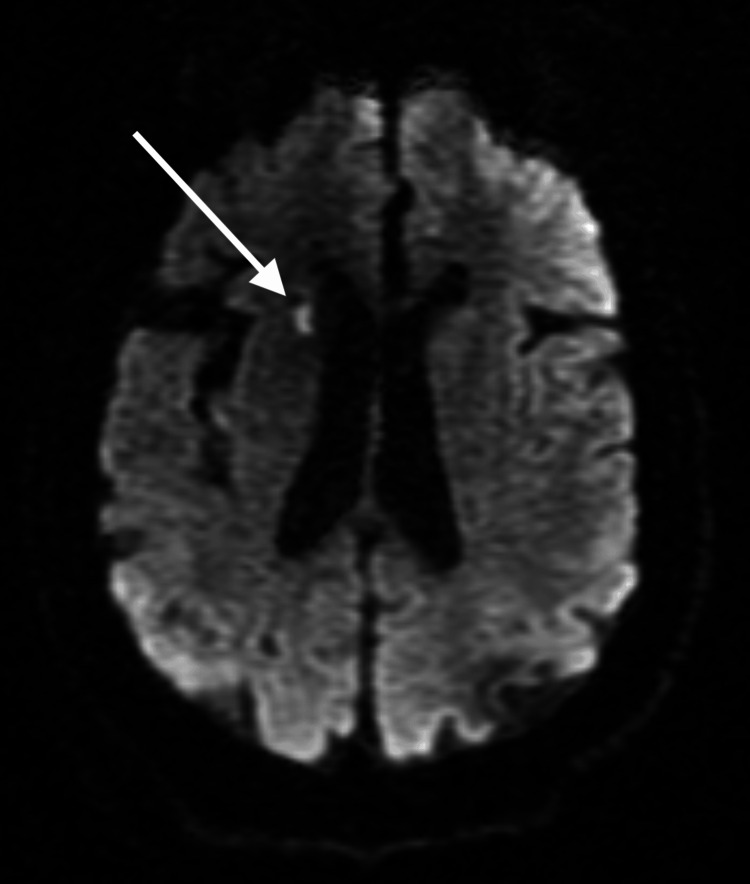
Diffusion-weighted T2 MRI showing infarct The arrow in this MRI scan points to an acute to subacute lacunar infarction in the right head of the caudate nucleus. The radiologist noted no acute processes that could explain the patient's encephalopathy.

The patient’s hospital course became complicated by somnolence and she had difficulty conversing with the team. The neurological exam at this time showed a decline. She was somnolent and only oriented to her name. She was arousable to loud stimuli, but couldn't follow commands. An MRI of the brain was done to evaluate for an acute cause, which later came back with acute to subacute lacunar infarction in the right head of the caudate nucleus (shown in Figure 1); however, her symptoms were less likely due to stroke and raised a higher suspicion of lupus cerebritis. A systematic review of symptoms of stroke showed that 56% were associated with motor deficits, 41% with speech deficits, 31% with mental status change, and 37% with confusion [5]. She was obtunded but didn’t have any focal neurologic deficits most commonly associated with a stroke. A CTA of the head and neck was negative for any active vasculitis. She began receiving empiric intravenous (IV) 500 mg of methylprednisolone twice per day. The patient responded well to the high-dose corticosteroids.

Given the patient's workup for acute encephalopathy covered the most common causes, it didn’t reveal a definitive explanation for encephalopathy. Her final diagnosis is thought to be acute encephalopathy most likely secondary to lupus cerebritis. Following high-dose steroids, she showed improvement over the next few days which is consistent with standard treatment for acute lupus cerebritis. As the patient’s somnolence improved following the steroid regimen, we are less likely to consider this due to hospital-acquired delirium.

## Discussion

Systemic lupus erythematosus (SLE) is a rare medical condition with a prevalence of 52.2 per 100,000 people. Cerebritis secondary to lupus is poorly classified with a range of 12-95% of patients with lupus experiencing neurological symptoms [4]. This is one of the reasons it is important to work up patients with lupus because it can have different presenting symptoms. It often can present with a wide variety of symptoms including but not limited to headache, mood disorders, cognitive malfunction, seizures, and cerebrovascular disease [6]. The case presented here shows the difficulty of clinically distinguishing between lupus cerebritis and other causes of acute encephalopathy.

Given the patient’s initial presentation of the vague symptoms, a complete workup for metabolic, toxic, drug, and infectious-related causes was needed even in the case of prior lupus cerebritis. The elevated troponins presented an emergent problem as acute coronary syndrome needed to be ruled out immediately. With this ruled out, the list of differentials for acute encephalopathy needed to be worked up. In this case, the patient's acute encephalopathy posed a challenge due to its symptomatic overlap between lupus cerebritis and other potential etiologies, such as infections, metabolic disturbances, medication non-compliance, or hypothyroidism.

Complement levels can be utilized to identify active flares in patients with lupus. As a result of the immune complex deposition and subsequent activation of the complement pathways, C3 and C4 are typically found to be decreased. One study found that this wasn’t always the case. A study discovered that a reduced C3 level had a sensitivity of 70%, while C4 had a sensitivity of 49% [7]. Our patient's C3 was slightly decreased with a normal C4 value, which isn’t a strong sign of an active lupus flare but doesn’t rule it out. Our results align with the literature, in that C3 has a higher sensitivity for active flare than C4. Another possible explanation for the small drop is an episode of non-inflammatory lupus. It is possible that a lack of an inflammatory response could cause the C3 not to drop as much as in an acute inflammatory lupus flare.

MRI/CT scans can be used for the identification of acute strokes as well as possible vasculitis secondary to acute lupus cerebritis. Generally, the MRI is a better test because it has a higher sensitivity however, it typically takes longer and costs more. A study demonstrated that MRI identified abnormal findings in 75% of lupus encephalopathy patients [8]. However, in our case, both the MRI and CT scans failed to show explicit evidence of active vasculitis. This aligns with the pattern of non-inflammatory lupus because of the subtle manifestations of the imaging. 

In the absence of positive test results and minimally elevated antinuclear antibodies (ANA) (1:160), our patient received high-dose (500 mg twice in a day (BID)) methylprednisolone for three days. This treatment proved to be effective in resolving the patient’s condition, leading to a return to her baseline. High-dose steroids are first-line treatments for active lupus flares. In other cases of neurological complications secondary to lupus, other case reports have shown that high doses for a brief duration helped alleviate neurological symptoms in patients with lupus encephalopathy [9]. The presence of very mild elevations in CRP and low titers, further points toward a non-inflammatory picture for this patient’s presentation.

The presented case involved a comprehensive workup encompassing various imaging studies and blood tests to rule out the most common causes of acute encephalopathy. This exhaustive diagnostic process, although important, delayed the treatment to pinpoint the precise cause. The financial cost to the patient and to the hospital is also something to consider as well, as a prolonged hospital stay can cause financial hardships for patients. Notably, the patient’s drastic improvement upon receiving the high-dose steroids raises the potential question of whether empirical treatment could be an option to be considered alongside the ongoing workup for other potential causes.

## Conclusions

In the absence of a clear etiology for acute encephalopathy, the patient with a prior history of lupus cerebritis was treated with high-dose steroids after a complete workup. The patient had a resolution of somnolence and confusion that occurred during the acute encephalopathy. Further research for lupus cerebritis should be directed toward identifying markers or improving diagnostic criteria for non-inflammatory lupus cerebritis to prevent delays in treatment.
